# Disparities in dialysis treatment and outcomes for Dutch and Belgian children with immigrant parents

**DOI:** 10.1007/s00467-012-2135-7

**Published:** 2012-03-21

**Authors:** Nikki J. Schoenmaker, Wilma F. Tromp, Johanna H. van der Lee, Brigitte Adams, Antonia H. Bouts, Laure Collard, Karlien Cransberg, Rita van Damme-Lombaerts, Nathalie Godefroid, Koen J. van Hoeck, Linda Koster-Kamphuis, Marc R. Lilien, Ann Raes, Jaap W. Groothoff

**Affiliations:** 1 Department of Pediatric Nephrology, Emma Children’s Hospital AMC Amsterdam, Amsterdam, The Netherlands; 2 Department of Pediatric Clinical Epidemiology, Emma Children’s Hospital AMC Amsterdam, Amsterdam, The Netherlands; 3 Department of Pediatric Nephrology, Hospital Université des Enfants Reine Fabiola Brussels, Brussels, Belgium; 4 Department of Pediatric Nephrology, Centre Hospitalier Universitaire de Liege, Liege, Belgium; 5 Department of Pediatric Nephrology, Sophia Children’s Hospital Erasmus MC Rotterdam, Rotterdam, The Netherlands; 6 Department of Pediatric Nephrology, University Hospital Leuven, Leuven, Belgium; 7 Department of Pediatric Nephrology, Université catholique de Louvain Brussels, Brussels, Belgium; 8 Department of Pediatric Nephrology, University Hospital Antwerp, University of Antwerp, Antwerp, Belgium; 9 Department of Pediatric Nephrology, University Medical Center St Radboud Nijmegen, Nijmegen, The Netherlands; 10 Department of Pediatric Nephrology, Wilhelmina Children’s Hospital University Medical Center Utrecht, Utrecht, The Netherlands; 11 Department of Pediatric Nephrology, University Hospital Gent, Gent, Belgium; 12 Academic Medical Centre, Dialysis Department, A01.247, Postbox 22660, 1100 DD Amsterdam, The Netherlands

**Keywords:** End-stage renal disease, Dialysis, Children with immigrant parents, Non-Western European, Renal osteodystrophy

## Abstract

**Background:**

In Belgium and the Netherlands, up to 40% of the children on dialysis are children with immigrant parents of non-Western European origin (non-Western). Concerns exist regarding whether these non-Western patients receive the same quality of care as children with parents of Western European origin (Western). We compared initial dialysis, post-initial treatment, and outcomes between non-Western and Western patients on dialysis.

**Methods:**

All children <19 years old on chronic dialysis in the Netherlands and Belgium between September 2007 and May 2011 were included in the study. Non-Western patients were defined as children of whom one or both parents were born in non-Western countries.

**Results:**

Seventy-nine of the 179 included patients (44%) were non-Western children. Compared to Western patients, non-Western patients more often were treated with hemodialysis (HD) instead of peritoneal dialysis (PD) as first dialysis mode (52 vs. 37%,* p* = 0.046). Before renal transplantation, non-Western patients were on dialysis for a median (range) of 30 (5–99) months, vs. 15 (0–66) months in Western patients (*p* = 0.007). Renal osteodystrophy was diagnosed in 34% of non-Western vs. 18% of Western patients (*p* = 0.028). The incidence rate ratio [95% confidence interval] for acute peritonitis was 2.44 [1.43-4.17] (*p* = 0.032) for non-Western compared to Western patients.

**Conclusions:**

There are important disparities between children on chronic dialysis with parents from Western European origin and those from non-Western European origin in the choice of modality, duration, and outcomes of dialysis therapy.

## Introduction

In the Netherlands and Belgium, a considerable proportion of children treated with chronic dialysis have immigrant parents from non-Western European origin (non-Western). Over the last decade, the proportion of non-Western immigrants in the Netherlands has grown from 9 to 11% [1]. Data on clinical management and outcomes of patients of ethnic minorities are scarce. Yet, there is a growing awareness that there are considerable healthcare disparities between immigrants and native American patients [2, 3]. Stronks et al. showed that in the Netherlands, the use of general practitioner care and the use of prescribed drugs has increased among non-Western adult patients as compared to adult patients from Western European (Western) origin [4].

Cultural misunderstandings between doctor and patient as well as language barriers may contribute to a different approach and subsequent disparities in quality of care. For example, a Dutch study on asthma in children showed ethnicity and limited Dutch proficiency to be important risk factors for uncontrolled asthma in children [5]. Disparities for patients of non-Western background may be even more important for highly complex modes of healthcare such as chronic renal replacement therapy in children, which makes great demands on the understanding and discipline of patients and caretakers.

Little is known about the management and outcomes of children of non-Western immigrant parents requiring chronic dialysis. We therefore compared the choice of initial dialysis modality, post-initial treatment, and outcomes between first- and second-generation children of non-Western origin and those from Western origin on chronic dialysis treatment.

## Methods

### Patients

All children aged < 19 years who were treated with hemodialysis (HD) or peritoneal dialysis (PD) in the Netherlands and Belgium between September 1, 2007 and May 1, 2011 were included. Data were collected as part of the RICH-Q project (Renal Insufficiency therapy in Children - Quality assessment and improvement) [6]. Follow-up data were available until May 1, 2011, or until transplantation, transition to adult care, or death. We obtained ethical approval from the ethical institutional review boards of all participating hospitals and written informed consent from the parents of all participants, and the participants themselves, if possible.

### Survey on preferred first dialysis therapy

At the start of RICH-Q in 2007 we asked one pediatric nephrologist per participating center to complete a questionnaire on treatment policies. The questionnaire was developed with input from all participating pediatric nephrologists to ensure content validity. The questionnaire included the following question: What is your preferred first mode of renal replacement therapy, if pre-emptive transplantation is not possible, in children > 3 years old?

### Data collection procedures

Data were collected from the medical records of the patients by trained local research nurses or by the participating pediatric nephrologists. The following data were used: age, gender, primary cause of end-stage renal disease (ESRD), date of first chronic renal replacement therapy (cRRT), first cRRT modality, waiting time on dialysis, country of birth for the child and parents, race/ethnicity as reported by the parents , i.e., Caucasian, Black, Asian, or other, and the educational attainment of the parents. Primary causes of ESRD were classified into seven categories: glomerulopathy, hemolytic uremic syndrome, urinary tract malformation, dysplasia, primary interstitial nephritis, tubular necrosis, or other. The following outcomes were recorded: blood values of hemoglobin, phosphate, calcium, immunoreactive parathyroid hormone (iPTH), alkaline phosphatase, homocysteine, presence of hypertension, medication prescriptions, and presence of metabolic bone disease at inclusion of the patients from 2007 until 2011. Complications of the therapy, such as peritonitis incidence, HD infections, and hospitalizations were monitored during the 4-year study period.

### Definitions

Non-Western patients were defined as children of whom one or both parents were born in non-Western European countries [1]. Western patients were defined as children from parents born in Western Europe. The total number of months on dialysis was defined as the period between the first day of dialysis and May 1, 2011 or the date of transplantation, the date of transition to adult care, or the date of death. For the calculation of the estimated glomerular filtration rate (eGFR), we used the updated version of the Schwartz formula: eGFR (ml/min/1.73 m^2^) = 0.413* (height in cm) / serum creatinine in mg/dl) [7]. The mean was calculated of three systolic and three diastolic blood pressure measurements at the time of inclusion. Hypertension was defined as either a systolic or diastolic blood pressure > p95 of the Task Force Report normal values corrected for age and gender [8]. Renal osteodystrophy (ROD) was diagnosed by an experienced radiologist based on a hand X-ray at the time of inclusion. We made a distinction between “incident patients”, who were included within 3 months after the start of dialysis, and “prevalent patients” defined as patients who had been treated with dialysis for at least 3 months before inclusion. “Poor condition as reason to start dialysis” was defined by the pediatric nephrologists. Parental educational attainment was defined as the highest educational level as reported by the parents themselves.

### Statistical analysis

Chi-square test or, in case of small expected cell counts, Fisher’s exact test, was used to test comparisons of categorical variables between non-Western and Western patients. Student’s* t* test and Mann–Whitney* U* test were used for continuous variables when appropriate.

Kaplan–Meier survival analysis was used to analyze waiting time on dialysis until transplantation. To investigate the association between non-Western status and number of peritonitis episodes per PD year at risk and ROD, respectively, linear and logistic regression analysis was performed. At first, only the central determinant was entered into the model (model with one determinant); possible confounders were then entered one at a time (multivariable models). If the regression coefficient of the central determinant ‘non-Western status’ changed >10% after the addition of one determinant to the regression model, this added determinant was considered to be a confounder and was kept in the final model. To evaluate the change in the regression coefficient, the same population was included in the bi-variable analysis as in the multivariable analysis, i.e., excluding any subjects with missing values.

The following determinants were considered as possible confounders: the duration of dialysis before inclusion, poor condition as the reason to start dialysis, the cause of ESRD, and educational attainment of parents.

All analyses were performed using SPSS 18.0 for Windows statistical software. For the incidence rate of infection and hospitalization, the number of new episodes per patient year at risk was calculated. The incidence rate ratio (IRR) was calculated to compare non-Western and Western patients. Hospitalization was analyzed separately in children < 4 years old, because the hospitalization rate is on average higher in young children.

## Results

### Characteristics and initial treatment

We included 179 children, of whom 79 (44%) had parents from non-Western origin (Table 1). In four patients (2%), only one parent was from non-Western origin; we considered these patients as ‘non-Western’ according to the definition of Statistics Netherlands (CBS) [1].

**Table 1:** Demographics, cause of end-stage renal disease (ESRD) and initial treatment of children with immigrant parents of non-western European origin (non-Western) and children with parents of western-European origin (Western)

	Non-Western,* n* = 79 (44)	Western,* n* = 100 (56)	*p* value
Male	50 (63)	52 (52)	0.130^a^
Origin of parents	Morocco: 15	Netherlands: 63	-
	Turkey: 15	Belgium: 34	
	Surinam: 9	Germany: 1	
	Asia: 5	Luxembourg: 1	
	Dutch Antilles/Caribbean: 3	United Kingdom: 1	
	Russia: 2		
	Africa (other): 15		
	Middle East (other): 15		
Country of birth	Netherlands: 37	Netherlands: 63	-
	Belgium: 13	Belgium: 34	
	United Kingdom: 2	Germany: 1	
	Morocco: 2	Luxembourg: 1	
	Turkey: 3	United Kingdom: 1	
	Surinam: 3		
	Asia: 2		
	Dutch Antilles/Caribbean: 2		
	Africa (other): 6		
	Middle East (other): 9		
Ethnicity			-
Caucasian	42 (53)	97 (97)	
Black	25 (32)	0	
Asian	7 (9)	0	
Mixed	5 (6)	3 (3)	
Primary cause of ESRD			0.787^c^
-Glomerulopathy	18 (23)	28 (28)	
-Hemolytic uremic syndrome	4 (5)	6 (6)	
-Urinary tract malformation	15 (19)	16 (16)	
-Dysplasia	20 (26)	24 (24)	
-Primary interstitial nephritis	1 (1)	4 (4)	
-Tubular necrosis	5 (6)	8 (8)	
Other	16 (20)	14 (14)	
Educational attainment of the parents			0.007^a^
-Elementary school	15 (19)	12 (12)+1.36	
-Secondary education	8 (10)	28 (28)+1.37	
-Post-secondary education missing	9 (11)	30 (30)+1.38	
-Missing	47 (60)	30 (30)+1.39	
Initial treatment of ESRD. Age at start of dialysis, years* median (range)			
Total	6.4 (0–17.0)	6.4 (0–18.5)	0.551^b^
HD	10.0 (0–17.0)	8.3 (1.1–18.5)	0.814^b^
PD	2.1 (0–16.6)	4.9 (0–16.7)	0.078^b^
eGFR (ml/1.73 m^2^) at start			
dialysis*	7.4 (3.5–20.5)	7.5 (2.4–31.5)	0.687^b^
“Poor condition” as reason to start dialysis	24 (30)	36 (36)	0.429^a^
First dialysis modality			0.046^a^
HD	41 (52)	37 (37)	
PD	38 (48)	63 (63)	
PD modality			0.241^a^
CCPD/APD	28 (78)	55 (82)	
CAPD	7 (19)	7 (11)	
Missing	1 (3)	5 (7)	
Inclusion in RICH-Q			
Prevalent patients	46 (58%)	63 (63%)	0.516^a^
Duration of dialysis before inclusion months*^1^			
HD (*n* = 42)	17.5 (3.8–63.4)	8.2 (3.1–65.3)	0.018^b^
PD (*n* = 46)	19.3 (4.1–77.5)	10.6 (3.4–49.4)	0.453^b^

Data are presented as* n* (percentage); *data are presented as median (range), ^a^Chi-square test, ^b^Mann–Whitney* U* test, ^c^Fisher’s exact test, ^1^From start of dialysis until inclusion for the prevalent patients),* eGFR* estimated glomerular filtration rate Schwartz. ( Ref. 2009),* HD* hemodialysis,* PD* peritoneal dialysis, *CCPD* continuous cycling peritoneal dialysis, *CAPD* continuous ambulatory peritoneal dialysis, *APD* automated peritoneal dialysis, *RICH-Q* renal insufficiency therapy in children-quality assessment and improvement. Prevalent defined as >3 months treated with dialysis before inclusion

We found no statistically significant differences between non-Western and Western patients with respect to primary cause of ESRD, gender, age at start of dialysis (HD or PD), eGFR at start of dialysis, poor condition as the reason to start dialysis, PD modality, or percentage of patients already treated with dialysis at the time of inclusion. Compared with Western children, non-Western children were significantly more often treated with HD as the first mode of cRRT, 37 vs. 52%, respectively (*p* = 0.046). The median duration of dialysis at the start of the RICH-Q project was 9 months longer in non-Western patients; in patients treated with HD, this difference was statistically significant (*p* = 0.018). Parents of non-Western European origin had a significantly lower level of educational attainment (Table 1).

### Preferred first dialysis therapy

Of the ten centers, six (60%) preferred PD as first dialysis treatment if pre-emptive transplantation was not possible for children > 3 years old. In the four centers preferring HD, this preferred mode of dialysis was more often followed in non-Western children than in Western children (*p* = 0.006).

### Post-initial dialysis treatment

Compared with Western patients, non-Western patients had a significantly longer median duration of HD, the median difference exceeding 10 months (*p* = 0.003) (Table 2). No significant disparities were found between the two groups for the median duration of PD, the number of switches in therapy, medication use, or the time on hemodialysis per week (Table 2). Relatively more non-Western than Western patients on HD had a Cimino fistula instead of a central venous catheter (53 vs. 33%), although this difference was not statistically significant (*p* = 0.080).

**Table 2:** Comparison of modes of dialysis and medication use between children with immigrant parents of non-western European origin (non-Western) and children with parents of western-European origin (Western)

Dialysis characteristics	Non-Western,* n* = 79	Western,* n* = 100	*p *value
Duration and mode of dialysis			
Total months on HD*^1^	16.8 (0.6–106.0)	6.5 (0.1-66.2)	0.003^b^
Total months on PD*^1^	11.4 (0.2–88.3)	13.6 (0.1–73.3)	0.756^b^
Duration of dialysis before first Tx*^2^ (months)* n* = 88	30.2 (5.0–99.0)	15.0 (0.0–66.7)	0.007^b^
Number of switches			
HD to PD	10	7	0.712^a^
PD to HD	14	15	0.307^a^
Total hours on HD per week*	12 (3-24)	12 (3-24)	0.670^b^
Vascular access			0.080^a^
- Cimino fistula	23 (53%)	11 (33%)	
- Central venous catheter	20 (47%)	22 (67%)	
Medication prescription			
Anti-hypertensive drugs	39 (52%)	61 (61%)	0.280^a^
> 2 anti-hypertensive drugs	14 (25%)	12 (29%)	0.603^a^
Erythropoietin	68 (91%)	92 (92%)	0.790^a^
Phosphate binder			
-Calcium based	39 (52%)	44 (44%)	0.359^a^
-Non-calcium based	39 (52%)	51 (51%)	1.000^a^
Etalpha	60 (80%)	81 (81%)	1.000^a^
Growth hormone	25 (33%)	33 (33%)	1.000^a^
Prophylactic antibiotics exit site	17 (47%)	22 (35%)	0.242^a^

Data are presented as* n* (percentage), * data are presented as median (range), ^a^Chi-square test, ^b^Mann–Whitney* U* test, ^1^From start of dialysis until May 1, 2011 or until transplantation, transition to adult care, or death. ^2^From start of dialysis until first transplantation before May 1, 2011,* HD* hemodialysis,* PD* peritoneal dialysis,* Tx* transplantation

**Fig. 1:**
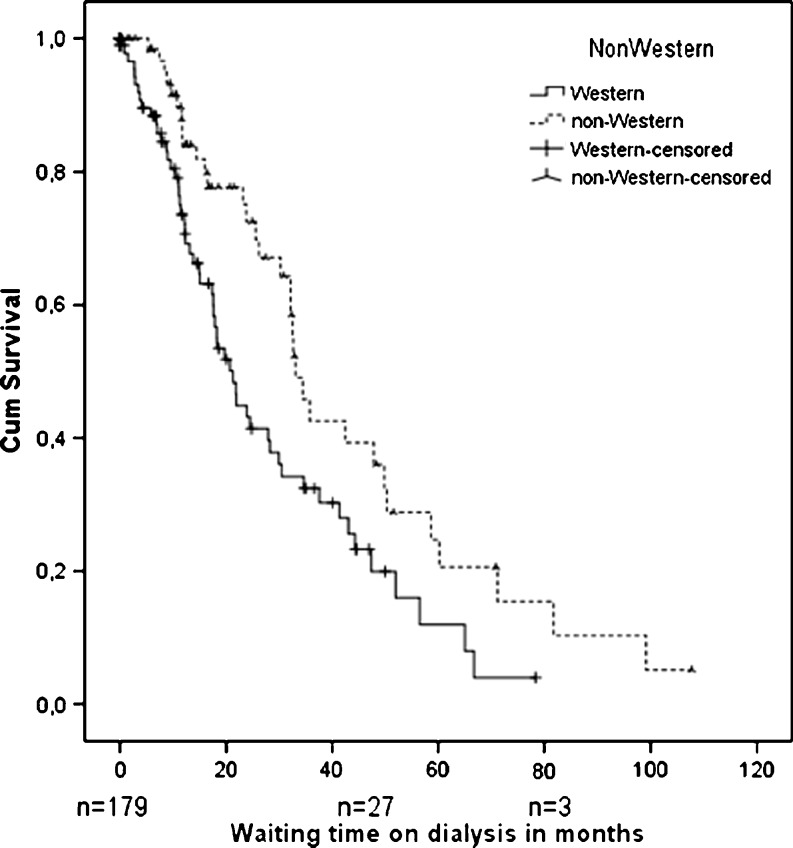
Time from start of dialysis to first transplantation in non-Western children compared to Western children,* p* = 0.007

During the follow-up period, 88 children (33 non-Western and 55 Western) received a kidney transplant. Prior to transplantation, non-Western patients spent a significantly longer time on dialysis than Western patients, with a median of 30 months versus 15 months, respectively (*p* = 0.007) (Fig. 1).

### Dialysis outcomes

The incidence rate of peritonitis was 1.0 per patient year at risk for non-Western and 0.41 per patient year at risk for Western patients. The incidence rates of HD exit site/tunnel infections were comparable in both groups (Table 3).

**Table 3:** Comparison of the outcomes between children with immigrant parents of non-western European origin (non-Western and children with parents of western-European origin (Western) with end-stage renal disease

	Non-Western		Western		*P* value	IRR [95%CI]
Blood values (at inclusion)	*n*	Median (range)	*n*	Median (range)		
Hemoglobin mmol/l						
-Incident	30	6.8 (4.1–8.7)	34	6.5 (3.6–8.2)	0.174^b^	
-Prevalent	46	6.9 (4.6–9.4)	60	7.0 (3.5–9.0)	0.871^b^	
Phosphate mmol/l						
-Incident	30	1.65 (1.02–3.16)	34	1.58 (0.82–2.96)	0.527^b^	
-Prevalent	46	1.68 (0.78–2.58)	60	1.68 (0.85–2.79)	0.831^b^	
Calcium mmol/l						
-Incident	28	2.44 (1.25–3.24)	33	2.40 (1.89–2.76)	0.767^b^	
-Prevalent	46	2.45 (2.00–3.09)	59	2.43 (1.90–3.58)	0.481^b^	
iPTH pmol/l						
-Incident	29	14.9 (0.1–159)	28	11.6 (0.1–121)	0.534^b^	
-Prevalent	44	17.6 (0.3–158)	58	14.1 (0.4–176)	0.570^b^	
Alkaline phosphatase U/l						
-Incident	8	411 (71– 987)	10	233 ( 4–825)	0.324^b^	
-Prevalent	22	408 (84–1,254)	24	201 (33–1,716)	0.043^b^	
Homocysteine umol/l						
-Incident	6	9.5 (4.2–14.2)	10	13.3 (5.3–30.7)	0.093^b^	
-Prevalent	19	10.6 (3.0–26.0)	29	12.1 (6.0–113.0)	0.217^b^	
Hypertension^1^						
(at inclusion)	76	33 (43)	4	45 (47)	0.607	
Renal osteodystrophy (at inclusion)						
X-ray hand: Moderate or severe signs						
Total	65	22 (34)	83	15 (18)	0.028^a^	
-incident	22	6 (27)	29	3 (11)	0.150	
-prevalent	43	16 (36)	54	12 (22)	0.120	
Infection						IRR [95%CI]
Number of peritonitis episodes per patient year PD at risk	34	1.00	60	0.41	-	2.44 [1.43–4.17]
Number of HD exit sites or tunnel infections per patient year HD at risk	46	0.34	33	0.45	-	0.75 [0.38–1.50]
Hospitalization						
Total number of hospitalizations per patient	79		100			
Year at risk		2.72		3.71	-	0.73 [0.62–0.87]
Age < 4 years		4.34		5.49		0.79 [0.60–1.03]
Age > 4 years		2.29		3.12		0.73 [0.59–0.91]
HD		1.97		2.56		0.77 [0.58–1.03]
PD		3.34		3.33		1.00 [0.79–1.26]
Days of hospitalization per year	73		88			
All ages		18 (0–282)		12 (0–196)	0.427^b^	
<4 years		35 (7–282)		22 (0–196)	0.228^b^	
>4 years		12 (0–162)		10 (0–158)	0.422^b^	

Data are presented as* n* (percentage), * data are presented as median (range), ^a^ Chi-square test, ^b^Mann–Whitney* U* test; *iPTH* immunoreactive parathyroid hormone, *HD* hemodialysis,* PD* peritoneal dialysis,* IRR* incidence rate ratio, 95% CI = 95% confidence interval. ^1^Hypertension was defined as systolic or diastolic blood pressures > p95 of the Task Force Report normal values corrected for age and gender [8].* Prevalent* is defined as >3 months treated with dialysis before inclusion.* Incident* is defined as included within 3 months after start of dialysis

The hospitalization rate in children older than 4 years was lower in non-Western than in Western patients. Children treated with PD were admitted to the hospital more often than children on HD. Within the subgroups of HD and PD, treatment modalities the hospitalization rates of non-Western and Western patients did not differ significantly. The number of days of hospitalization was not significantly different between the groups either above or below the age of 4 years.

Compared to Western patients, non-Western patients had more peritonitis episodes, the mean difference [95% CI] being 0.58 [0.03–1.14] episodes per year at risk, adjusted for poor condition as the reason to start dialysis (Table 4a).

**Table 4:** Results of linear regression analysis for episodes of peritonitis/PD year at risk in 94 children treated with peritoneal dialysis in all PD patients (5a) and in all patients in whom also data on the parental educational attainment was obtained (5b)

4a	B	95% CI
Model with one determinant (*n* = 94)		
Non-Western status	0.65	0.09–1.21
Multivariable models		
1. Non-Western status	0.63	0.72–1.19
- Months on PD before inclusion	–0.01	–0.09 – 0.08
2. Non-Western status	0.58	0.03–1.14
-“Poor condition” as reason to start	–0.60	(–1.15) – (–0.05)
		
4b		
Model with one determinant (*n* = 60)		
Non-Western status	0.93	0.11–1.74
Multivariable models		
1. Non-Western status	0.79	–0.04 – 1.62
-“Poor condition” as reason to start	–0.55	–1.32 – 0.22
2. Non Western status	0.72	–0.13 – 1.56
-“Poor condition” as reason to start	–0.52	–1.30 – 0.27
Parental educational attainment (reference category: “post secondary” )		
-Elementary school	0.33	–0.59 – 1.25
-Secondary education	–0.14	–0.96 – 0.67

*PD* peritoneal dialysis

Signs of ROD at inclusion were present in 22 non-Western (34%) and 15 Western patients (18%) (*p* = 0.028) (Table 3). ROD was present before the start of dialysis in 27% and 11% of the non-Western and Western patients, respectively. When adjusted for parental educational attainment, the OR [95% CI] for ROD in non-Western patients was 1.8 [0.6–5.5] (Table 5b).

**Table 5:** Results of logistic regression analysis for renal osteodystrophy in 148 children treated with dialysis (5a) and in a subgroup of 87 patients on dialysis of whom we obtained data on educational attainment of the parents (5b)

5. a	OR	95% CI
Model with one determinant (*n* = 148)		
Non-Western status	2.32	1.09–4.96
Multivariable models		
1. Non-Western status	2.25	1.05–4.82
Months on dialysis before diagnosed with ROD	1.00	1.00–1.00
2. Non-Western status	2.33	1.06–5.11
Cause of ESRD (reference category: “other”)		
-Glomerulopathy	0.57	0.19–1.76
-Hemolytic uremic syndrome	0.27	0.03–2.57
-Urinary tract malformation	0.40	0.10–1.59
-Dysplasia	0.66	0.21–2.01
-Primary interstitial nephritis	1.12	0.83–14.92
-Tubular necrosis	1.55	0.33–7.36
3. Non-Western status	2.46	1.14–5.34
“Poor condition” as reason to start	2.13	0.97–4.63
		
5. b		
Model with one determinant (*n* = 87)		
Non-Western status	2.41	(0.85–6.80)
Multivariable model		
1. Non-Western status	1.75	(0.56–5.47)
Educational attainment of parents (reference category: “post-secondary” )		
-Elementary school	4.88	(1.20–19.77)
-Secondary education	2.33	(0.61–8.94)

*ESRD* end-stage renal disease

Six children (3%) died during the follow-up period, three non-Western and three Western patients. Four children (two in each group) died of intercurrent diseases, one of Wilms’ tumor, one of congenital heart disease, and two due to withdrawal of treatment because of multiple co-morbidities. Two children (one in each group) died of complications of the treatment of the underlying kidney disease, one of pulmonary hypertension, and one due to a complication of a central line.

## Discussion

We found less favorable health outcomes and important disparities in the management of children on dialysis treatment with parents from non-Western European origin as compared to those from Western origin in the Netherlands and Belgium. We will discuss the possible reasons for these disparities and formulate areas for further research.

In both countries, non-Western patients more often received HD as the first mode of cRRT instead of PD as compared to Western children. Although kidney transplantation is considered to be the optimal mode of cRRT for children with ESRD, in most circumstances dialysis treatment as first cRRT is inevitable [9]. There is no consensus on the optimal mode of dialysis treatment. Peritoneal dialysis (PD) is offered to younger children, especially under the age of 2 years or weighing less than 10 kg. Some centers recommend PD instead of HD in children because of its social advantages [10]. PD permits a relatively normal lifestyle by allowing children to attend school full time with less dietary and fluid restrictions compared to HD. In addition, obtaining vascular access for HD in children can be challenging [11]. In some European countries, hemodialysis (HD) is preferred for children over the age of 5 years [12]. Factors that are reported to influence the choice of therapy include the age of the child (30%), parent preference (27%), distance from the unit (14%), patient preference (11%), social condition (7%), and inability to do one particular mode (6%) [12, 13]. In our study, 40% of the centers preferred HD for children > 3 years old. We found that in Western children, the preferred mode of dialysis according to the center policy was less often followed than in non-Western children. If the preferred mode of dialysis was not followed in Western children, PD was chosen more often than HD. In non-Western children, HD prevailed if the preferred modality was not followed.

It is not clear whether the deviations from the center policy had merely been the physicians’ or the patients’ choice. It is possible that Western parents more often prefer a situation of having the child out of the hospital as much as possible, as with PD, despite the burden of home therapy, even in a situation where the physician advises differently. Physicians, on the other hand, may have been reluctant to offer a home dialysis program to some non-Western patients. The preference for HD might even be due to a more systematic physician bias in the treatment of non-Western patients. Our findings are consistent with race-associated disparities in the dialysis treatment of children in the United States. Furth et al. found more use of HD instead of PD in children of African American descent. Family, patient, and provider preferences all accounted for the difference in choice of therapy by race [14]. In addition, they suggest that systematic racial bias specifically may have contributed to the difference in dialysis modality choice. The extent to which subtle or overt and systematic provider preconceptions about race have affected dialysis modality choice in the children in our study is unclear.

Financial motives may also play a role in the choice of either HD or PD. In the United States, unequal access to optimal medical care is an important cause for disparities in care. For example, Young et al. describe that pediatricians are facing the dilemma of rationing care to uninsured, undocumented children, especially for expensive life-saving care such as renal transplantation and dialysis [15]. However, unlike in the United States, the social system in both the Netherlands and Belgium provides few financial incentives for choosing a certain treatment modality. Health insurance is mandatory. All insured patients pay an income-dependent contribution. In return, they receive all necessary medical and paramedical care for free, including dietary, psychological, and educational support. In the Netherlands, patients with a chronic disease can apply for a personal budget for extra help at home, depending on income and family situation. Even immigrants or refugees who are staying in the Netherlands or Belgium undocumented receive all necessary medical and paramedical care, if necessary for free [16]. In 2010, the Netherlands was ranked first in a study comparing the health care systems of the US, Australia, Canada, Great Britain, Germany, and New Zealand [17]. The study assessed five measures of healthcare; quality, efficiency, access to care, equity and, the ability to lead long, healthy, productive lives.

Non-Western patients spent significantly more time on dialysis prior to transplantation. The transplantation process involves a series of steps related to medical suitability, pre-transplant work-up, the possibility of a family member to donate, or movement up the waiting list for deceased donor to eventual transplantation [18]. Some of these steps have been examined individually in previous studies in the United States [19, 20]. The study of Ozminkowski et al. showed that in the United States, blacks move up the waiting list at a slower rate than whites [19]. Movement on the waiting list has been studied extensively, and appears to reflect both biological factors (e.g., HLA-based tissue typing) and non-biological factors (e.g., transplant center characteristics [20]). Furth et al. reported racial disparities regarding access to the renal transplant waiting list in pediatric units in the United States [21]. Whether these disparities were attributable to differences in the timing of presentation to a nephrologist, physician bias in identification of transplant candidates, or patient preferences was not clear.

The longer time on dialysis in our non-Western children could partly be explained by a different mode of transplantation. In an earlier study on the same cohort on transplantation policy and outcomes in non-Western children with end-stage renal disease, we found that non-Western patients less often received donor kidneys from a living related donor than deceased donor (DD) transplantations [22], contrary to Western patients.

Non-Western patients who were treated with PD more often had peritonitis episodes than Western patients. This is remarkable given the relatively small number of non-Western patients on PD as compared to HD, which suggests a positive selection for this modality. The incidence rates of HD exit site/tunnel infections were similar in both groups, but non-Western patients more often had a Cimino fistula. This might be related to different policies followed in non-Western and Western patients with respect to vascular access. As discussed before, non-Western patients were less often scheduled for living-related donor transplantation and consequently more often prepared for deceased donor transplantation with a longer expected time on HD. This may have motivated physicians to choose for a Cimino fistula more often in non-Western patients.

The hospitalization rate was lower in non-Western patients > 4 years than in Western patients of the same age. This might be explained by the fact that relatively more non-Western patients receive HD than Western patients. Children on HD are monitored regularly and intravenous antibiotics can be given during the HD treatment sessions, both circumstances that make admission less often necessary than in PD. Consistent with this, both non-Western and Western patients on PD were equally more often admitted than patients on HD.

We found that ROD was more prevalent in non-Western children. Nevertheless, in a multivariable analysis of a smaller subgroup without missing values, the educational attainment of the parents turned out to be a confounder of this association. Renal osteodystrophy (ROD), which is associated with a long duration of chronic kidney disease, is a major clinical problem in young adults with pediatric ESRD [23]. To detect ROD, a bone biopsy remains the 'gold standard' investigation. Its invasive nature and the need for specialized processing and interpretation limits the use of bone biopsy in clinical practice [24]. Hand X-ray is not invasive and has a high specificity (92%) for the detection of ROD bone features [25].

ROD was already present before the start of dialysis in six non-Western and three Western children. We have no evidence that non-Western patients were less compliant with therapy before they developed ESRD. We found no indication of non-adherence in surrogate outcome markers for prevention of ROD such as iPTH, phosphate and medication dosage of phosphate binders, and vitamin D derivates. Therefore, genetic factors influencing for instance the vitamin D status might have been involved. Prevention of ROD requires extreme discipline to strictly follow the dietary and medication regime. The combination of impaired schooling of the parents and cultural misunderstandings may have hampered compliance in some children of non-Western background. These patients might profit from a culturally tailored approach from the treatment team [26].

In the Netherlands, there has been a shift in the non-Western population. Especially in children, we see a new generation from non-Western origin. They were born in the Netherlands, and are mostly the second generation of non-Western descent whose behavior differs from elderly first-generation non-Western adults.

Perhaps this might be one of the explanations for the difference between the results of the present study and those of a recent Dutch study in adult immigrants that showed increased survival for adult immigrant patients on dialysis in the Netherlands compared to native Dutch dialysis patients [27]. Another explanation might be that ESRD in adult immigrant patients is due to different causes than in young patients.

A limitation of this study is the exclusion of children who had been transplanted before the start of the RICH-Q registry. The inclusion of patients in the RICH-Q project started in September of 2007. Only children that were treated with dialysis were included in the present study. Children who had been transplanted before this date were not included. This probably has resulted in an overrepresentation of children who are not suitable for (pre-emptive) transplantation, which may have resulted in a relatively large proportion of non-Western patients in this study. Therefore, the percentage of non-Western patients in this study is higher than in the general population. Another limitation is that we did not collect information on language barriers, which have been shown to impact a wide variety of health and healthcare outcomes [28]. The parental educational attainment seemed to be a confounder in the development of ROD. Unfortunately, information on the educational attainment of the parents was missing in the majority of non-Western European children.

In conclusion, children with end-stage renal disease with parents of non-Western European origin more often receive HD instead of PD as first cRRT modality, and stay on dialysis longer before transplantation. These non-Western patients have less favorable outcomes, such as an increased risk for PD-associated peritonitis and renal osteodystrophy. This situation might be improved by education of treatment providers about these disparities and about the potential roles of conscious and unconscious bias, cultural misunderstandings, and language barriers.

## References

[1] Centraal Bureau voor de Statistiek (2011) http://www.cbs.nl. Date accessed 1-12-2011

[2] Flores G, Olson L, Tomany-Korman SC (2005). Racial and ethnic disparities in early childhood health and health care. Pediatrics.

[3] Lanting LC, Bootsma AH, Lamberts SW, Mackenbach JP, Joung IM (2008). Ethnic differences in internal medicine referrals and diagnosis in the Netherlands. BMC Publ Health.

[4] Stronks K, Ravelli AC, Reijneveld SA (2001). Immigrants in the Netherlands: equal access for equal needs?. J Epidemiol Community Health.

[5] van Dellen QM, Stronks K, Bindels PJ, Ory FG, Bruil J, van Aalderen WM (2007). Predictors of asthma control in children from different ethnic origins living in Amsterdam. Respir Med.

[6] Tromp WF, van der Lee JH, Offringa M, Bouts AH, Collard L, Cransberg K, Van Damme-Lombaerts R, Godefroid N, Van HK, Koster-Kamphuis L, Lilien MR, Raes A, Groothoff JW (2011). Lessons learned from efforts to improve the quality of care in children with end-stage renal disease in the Netherlands and Belgium. Arch Dis Child.

[7] Schwartz GJ, Munoz A, Schneider MF, Mak RH, Kaskel F, Warady BA, Furth SL (2009). New equations to estimate GFR in children with CKD. J Am Soc Nephrol.

[8] Update on the 1987 Task Force Report on High Blood Pressure in Children and Adolescents: a working group report from the National High Blood Pressure Education Program. National High Blood Pressure Education Program Working Group on Hypertension Control in Children and Adolescents (1996) Pediatrics 98:649–658 8885941

[9] Boehm M, Winkelmayer WC, Arbeiter K, Mueller T, Aufricht C (2010). Late referral to paediatric renal failure service impairs access to pre-emptive kidney transplantation in children. Arch Dis Child.

[10] Tsai HL, Yang LY, Chin TW, Wang HH, Liu CS, Wei CF, Chang JW (2010). Outcome and risk factors for mortality in pediatric peritoneal dialysis. Perit Dial Int.

[11] Chan KS, Keeler E, Schonlau M, Rosen M, Mangione-Smith R (2005). How do ethnicity and primary language spoken at home affect management practices and outcomes in children and adolescents with asthma?. Arch Pediatr Adolesc Med.

[12] Fischbach M, Terzic J, Menouer S, Provot E, Bergere V (2001). Hemodialysis in children: principles and practice. Semin Nephrol.

[13] Fischbach M, Edefonti A, Schroder C, Watson A (2005). Hemodialysis in children: general practical guidelines. Pediatr Nephrol.

[14] Furth SL, Powe NR, Hwang W, Neu AM, Fivush BA (1997). Racial differences in choice of dialysis modality for children with end-stage renal disease. Pediatrics.

[15] Young J, Flores G, Berman S (2004). Providing life-saving health care to undocumented children: controversies and ethical issues. Pediatrics.

[16] Hong HW (2009) Somali Immigrants and Health Care: Neo-Liberal Globalization in the United States and Holland. 22 ed. http://digitalcommons.macalester.edu/macintl/vol22/iss1/11/

[17] Davis K, Schoen S, Stremikis K (2010) Mirror, Mirror on the Wall: How the Performance of the U.S. Health Care System Compares Internationally, 2010 Update. http://www.commonwealthfund.org/Publications/Fund-Reports/2010/Jun/Mirror-Mirror-Update.aspx?page=all

[18] Alexander GC, Sehgal AR (1998). Barriers to cadaveric renal transplantation among blacks, women, and the poor. JAMA.

[19] Ozminkowski RJ, White AJ, Hassol A, Murphy M (1997). Minimizing racial disparity regarding receipt of a cadaver kidney transplant. Am J Kidney Dis.

[20] Sanfilippo FP, Vaughn WK, Peters TG, Shield CF, Adams PL, Lorber MI, Williams GM (1992). Factors affecting the waiting time of cadaveric kidney transplant candidates in the United States. JAMA.

[21] Furth SL, Garg PP, Neu AM, Hwang W, Fivush BA, Powe NR (2000). Racial differences in access to the kidney transplant waiting list for children and adolescents with end-stage renal disease. Pediatrics.

[22] Tromp WF, van der Lee JH, Offringa M, Bouts AH, Collard L, Cransberg K, Van Damme-Lombaerts R, Godefroid N, Van HK, Koster-Kamphuis L, Lilien MR, Raes A, Groothoff JW (2012) Fewer pre-emptive renal transplantations and more rejections in immigrant children compared to native Dutch and Belgian children. Nephrol Dial Transplant. doi:10.1093/ndt/gfr628 10.1093/ndt/gfr628 22323533

[23] Groothoff JW, Offringa M, Van Eck-Smit BL, Gruppen MP, Van De Kar NJ, Wolff ED, Lilien MR, Davin JC, Heymans HS, Dekker FW (2003). Severe bone disease and low bone mineral density after juvenile renal failure. Kidney Int.

[24] Roe S, Cassidy MJ (2000). Diagnosis and monitoring of renal osteodystrophy. Curr Opin Nephrol Hypertens.

[25] Fletcher S, Jones RG, Rayner HC, Harnden P, Hordon LD, Aaron JE, Oldroyd B, Brownjohn AM, Turney JH, Smith MA (1997). Assessment of renal osteodystrophy in dialysis patients: use of bone alkaline phosphatase, bone mineral density and parathyroid ultrasound in comparison with bone histology. Nephron.

[26] Rodrigue JR, Cornell DL, Lin JK, Kaplan B, Howard RJ (2007). Increasing live donor kidney transplantation: a randomized controlled trial of a home-based educational intervention. Am J Transplant.

[27] van den Beukel TO, Dekker FW, Siegert CE (2008). Increased survival of immigrant compared to native dialysis patients in an urban setting in the Netherlands. Nephrol Dial Transplant.

[28] Flores G (2005). The impact of medical interpreter services on the quality of health care: a systematic review. Med Care Res Rev.

